# Disruption of intraflagellar protein transport in photoreceptor cilia causes Leber congenital amaurosis in humans and mice

**DOI:** 10.1186/2046-2530-1-S1-P83

**Published:** 2012-11-16

**Authors:** K Boldt, DA Mans, J Won, J van Reeuwijk, Y Texier, PM Nishina, R Roepman, M Ueffing

**Affiliations:** 1 Medical Proteome Center, Institute for Ophthalmic Research, University of Tübingen, Germany; 2 Division of Experimental Ophthalmology and Medical Proteome Center, Center of Ophthalmology, University of Tübingen, Germany; 3 Department of Human Genetics, Nijmegen Centre for Molecular Life Sciences, and Institute for Genetic and Metabolic Disease, Radboud University Nijmegen Medical Centre, the Netherlands; 4 The Jackson Laboratory , ME, USA

## 

Leber Congenital Amaurosis (LCA) is one of the most common forms of congenital blindness in young children. It is the most severe retinal dystrophy, characterized by strong visual impairment or blindness within the first months after birth. Despite its genetic heterogeneity, the clinical phenotype is highly overlapping, pointing towards a common disease mechanism. To gain insights into the mechanism, we compared the interactomes of wild type and LCA causing mutants of lebercilin by quantitative mass spectrometry using SILAC followed by MaxQuant based analysis. This revealed that the link of lebercilin to the IFT complex is disrupted due to LCA-associated mutations. The interaction of the IFT proteins with lebercilin was confirmed by GST pull-down from retina, the loss of IFT proteins from the complex of mutants by western blot. While the disruption of the lebercilin-IFT interaction does not lead to disturbed ciliogenesis in both, hTERT-RPE1 cell and in the generated lebercilin knockout mice, the light dependent translocation of arrestin and transducing is clearly impaired, as well as the transport of opsins to the outer segments of photoreceptors. As a consequence this results in improper outer segment formation and finally photoreceptor degeneration. These data suggest that the interaction of lebercilin with the IFT complexes is important for ciliary transport in photoreceptors and thereby for formation and/or maintenance of outer segments. Further, our findings demonstrate that the disruption of ciliary transport to the outer segment is a cause for LCA.

http://www.eye.uni-tuebingen.de/medical-proteome-center

